# The Effect of Different Processing Methods on Metabolite Profiles by Comparative Metabolomics in Kernels and Sprouted Seeds of Foxtail Millet

**DOI:** 10.3390/foods14111900

**Published:** 2025-05-27

**Authors:** Lingda Han, Qi Li, Xiaowen Wang

**Affiliations:** College of Food Science and Engineering, Shanxi Agricultural University, Taigu, Jinzhong 030801, China; lingda2004@163.com (L.H.); sxauliqi1992@outlook.com (Q.L.)

**Keywords:** millet, nutrients, germination, hot air drying, freeze-drying

## Abstract

Foxtail millet attracts much attention for its rich nutrients and health benefits. However, ultra-polishing has greatly reduced its nutrition. Germination can enhance nutrition value. Nevertheless, knowledge of nutrient changes in kernels and sprouted grains under different polishing methods and at different germination stages is limited. Here, comparative metabolomics was used to detect metabolite changes in differently polished millets (Manually Polished Millet, MPM; Ultra-Polished Millet, UPM; Manually Ultra-Polished Millet, MUPM) and in sprouted grains with hot air drying (HAD) and freeze-drying (FD) at different germination times. Compared to whole grains, MPM, UPM, and MUPM had 306 to 720 down-regulated metabolites, reducing most antioxidants, essential amino acids, fatty acids, and vitamins in whole grains. For sprouted grains, metabolic activities were comprehensively activated. The early stages accumulated basic nutrients such as free and functional amino acids, small sugars, and essential fatty acids. The 16 h stage increased secondary antioxidant metabolites like flavonoids, and the 24 h germination generated more functional components such as sulfur-containing metabolites. More basic nutrients were preserved by FD in comparison to the reduced basic nutrients and increased antioxidant accumulation associated with HAD. This work systematically characterizes the metabolite changes in polished millets and sprouted grains, providing a reference for developing functional millet products.

## 1. Introduction

Foxtail millet (*Setaria italica*), one of the oldest cultivated cereals, has been a staple in Asia and Africa for over 8000 years, especially in arid regions because of its remarkable drought tolerance [1]. Moreover, in ancient Chinese agriculture, it served as a primary grain, laying the foundation for early agrarian societies. Today, it remains a culturally significant crop, valued for its bioactive compounds and health benefits. Nutritionally, foxtail millet exhibits distinct metabolite profiles across its husk, bran, and kernel, underscoring its multifunctional value [2]. Millet-derived peptides demonstrate ACE-inhibitory activity linked to antihypertensive effects [3], while its lipid extracts show metabolic regulatory capacity through gut microbiota and serum metabolite modulation [4]. The functional properties of prolamin, millet’s core protein, enable texture enhancement and stability optimization in food products [5]. This is exemplified by the enhanced nutritional and textural quality of noodles when millet is combined with green banana flour [6]. Additionally, its protein concentrates and balanced amino acid profiles further contribute to its nutritional value, benefitting both protein deficiencies and dietary balance [7,8]. These attributes, combined with its robust antioxidant capacity and adaptability [9], position foxtail millet as a vital gluten-free crop, offering sustainable solutions to global malnutrition and chronic diseases.

However, modern dietary habits have prioritized ultra-polished millet products, leading to bran discard during dehusking and a loss of critical nutrients. Unlike dehusked and polished grains, whole grain retains the bran layer, offering a sustainable solution to reduce food waste while preserving nutritional integrity. In fact, foxtail millet bran is a nutrient-dense fraction rich in bioactive compounds. Its ethanol extracts, fiber, and bound polyphenols demonstrate antioxidant and anti-inflammatory properties [10,11,12]. Mechanistically, bran components alleviate hepatic injury [13], inhibit colorectal cancer through apoptosis and ROS modulation [14,15,16], and enhance chemosensitivity via STAT3 pathway blockade [16,17]. Additional benefits include improved metabolic regulation, gut microbiota modulation (particularly Lactobacillus), and immune enhancement through phenolic-bound arabinoxylans [18,19,20]. Retaining bran during processing maximizes these synergistic health benefits.

Germination is a transformative biochemical process that elevates seed nutrition by activating enzymatic pathways, degrading anti-nutritional factors, and synthesizing bioactive compounds [21]. This metabolic reprogramming enhances both nutrient quality and bioavailability, positioning sprouted grains as nutrient-dense functional foods. Germination drives the accumulation of essential nutrients critical for human health. A notable shift is the surge in γ-aminobutyric acid (GABA), a neuroprotective amino acid synthesized by glutamate decarboxylase. This process simultaneously boosts levels of other amino acids (e.g., lysine and arginine) and enhances the bioavailability of iron and zinc, addressing common micronutrient deficiencies [22]. In legumes and pseudocereals like quinoa, sprouting also degrades protease inhibitors and storage proteins, converting them into digestible peptides that improve protein quality and reduce gastrointestinal irritation, benefits accompanied by a threefold increase in B-vitamins like thiamine and folate [23,24,25]. Secondary metabolism is equally reshaped during germination, with phenolic compounds, flavonoids, and benzoxazinoids rising significantly in cereals such as wheat and millet. Genetic variability influences these responses; for example, hard wheat varieties exhibit stronger antioxidant activity post-germination than soft ones, while sacha inchi seeds show enhanced phenolic content alongside altered flavor profiles [26,27]. Moreover, sprouted grains offer functional benefits through dietary fiber and polyphenol enrichment, as well as their porous structure, supporting gut microbiota balance and metabolic regulation [28,29].

Drying is a pivotal post-harvest step that shapes the nutritional and functional quality of millet products, especially for germinated grains abundant in heat-sensitive bio-actives. Among various drying methods, hot air drying (HAD) and freeze-drying (FD) are widely employed. Heated air removes moisture and risks protein aggregation and degrading heat-sensitive nutrients like phenolic acids and vitamins [30,31,32]. Operating at low temperatures, FD preserves heat-labile compounds (e.g., polyphenols and GABA) and structural integrity, retaining over 90% of antioxidant activity in sprouted grains [33]. Its porous matrix maintains solubility in proteins and volatile aromas in extracts, ideal for nutraceuticals, despite higher costs [32,34].

Despite growing insights into the nutritional benefits of whole millet and the metabolic enhancements of sprouted grains, our understanding of how specific polishing methods (e.g., manual vs. ultra-polishing) and germination stages impact nutrient composition and bioactive profiles remains fragmented. While studies in crops like wheat [26,35], quinoa [23,34], maize [36], rice [37], sacha inchi [27], and buckwheat [38] have utilized metabolomics to dissect germination-induced metabolic shifts or processing-related nutrient losses, comparable data for foxtail millet, especially across diverse polishing intensities and germination timepoints, is lacking. Metabolomics, a powerful tool for unbiased profiling of small-molecule metabolites, offers a unique opportunity to systematically characterize these changes. In this study, we employed metabolomics to investigate metabolite changes in differently polished millet (Manually Polished Millet, MPM; Ultra-Polished Millet, UPM; Manually Ultra-Polished Millet, MUPM) and in sprouted grains with hot air drying (HAD) and freeze-drying (FD) at different germination times (8 h, 16 h, and 24 h). The aims of this study are to: (a) Identify the metabolites in millets (whole grains, MPM, UPM, and MUPM) and sprouted grains with hot air drying (HAD) and freeze-drying (FD) at different germination times (8 h, 16 h, and 24 h); (b) Screen for the differentially accumulated metabolites in millets or grains caused by different processing methods; (c) Clarify the characteristics of metabolite accumulation in seeds at different germination stages; (d) Compare the impacts of HAD and FD on the metabolites in sprouted grains. This study offers more understanding of the health benefit of whole grain and germinated seed in foxtail millet, and provides a reference for developing functional millet products.

## 2. Materials and Methods

### 2.1. Sample Preparation and Seed Germination

The foxtail millet cultivar “Changsheng 7” used in this study was grown in Qin Xian (36°25′ N, 112°29′ E, altitude 1150 m), Shanxi Province, China, in 2023, with average sunshine duration of 2475–2887 h, mean annual temperature of 22.6 °C, absolute temperature difference of 18.9 °C, annual precipitation 606 mm, evapotranspiration 1756.9 mm, and mean relative humidity 60.0%. The grains were stored in a Shanxi Qinzhou Huang Millet (Group) Co., Ltd. (Qinxian, China) granary at 4 °C until use. The experiment was carried out between September 2024 to January 2025. Three polishing methods were used to investigate nutritional changes in manually-polished, ultra-polished, and manually ultra-polished millet. Manually-polished and manually ultra-polished (termed “manual” in comparison with industry-based processing) were performed using a bench top husker machine JLGJ-45 (Ningbo, China) (based on physical pressure produced between two rolling bars) by adjusting dehulling intensity: three dehulling cycles were applied to construct MPM (Manually Polished Millet), and eight cycles plus gentle grinding with mortar and pestle for two minutes were used to generate MUPM (Manually Ultra-Polished Millet). In contrast, UPM (Ultra-Polished Millet) was prepared using “Changsheng 7” millet industrially refined by the Shanxi Qinzhou Huang Millet (Group) Co., Ltd. All samples, including dry seeds, MPM, UPM, and MUPM, were ground into powder using a grinder (MM 400, Retsch) at 30 Hz for 1.5 min for non-targeted metabolomics detection. Three replicates were set for each group.

For the germination experiment, a total of 200 g of seeds were used. First, shrunken seeds were removed, and the remaining seeds were rinsed four times with distilled water to eliminate surface dust and impurities. The seeds were then spread on a sterilized seedling tray, with sufficient water maintained at the tray bottom to ensure a moist environment. The tray was placed in an incubator set at 30 °C and 80% humidity. Germinated seeds were harvested at 8 h, 16 h, and 24 h. After blotting off surface moisture, samples at each stage were divided into two portions for freeze-drying (FD) and hot air drying (HAD) treatments. The FD group was freeze-dried under vacuum in a freeze-dryer (Model Scientz-100F, Ningbo Scientz Biotechnology Co., Ltd., Ningbo, China) for 63 h, then ground into powder using a grinder (Model MM 400, Retsch, Düsseldorf, Germany) at 30 Hz for 1.5 min. The HAD group was dried in a 60 °C hot air oven for 24 h before being ground into powder using the same method. Each sample was named according to the processing method and germination time. The FD group included FDS-8 h, FDS-16 h, and FDS-24 h (Freeze-Dried Seed), and the HAD group included ODS-8 h, ODS-16 h, and ODS-24 h (Oven-Dried Seed). Each subgroup had three replicates.

### 2.2. Nontargeted Metabolomics Detection

#### 2.2.1. Metabolite Extraction

Fifty milligrams of sample powder were weighed using an MS105DΜ electronic balance (Mettler-Toledo International Inc., Zurich, Switzerland) and transferred to a centrifuge tube, followed by the addition of 1200 μL of −20 °C pre-cooled 70% methanolic aqueous solution containing the internal standard. The mixture was vortexed for 30 s every 30 min, repeated six times to ensure thorough extraction. After centrifugation at 12,000 rpm for 3 min, the supernatant was carefully aspirated, filtered through a 0.22 μm microporous membrane, and transferred to an injection vial for subsequent UPLC-MS/MS analysis.

#### 2.2.2. High-Performance Liquid Chromatography-Mass Spectrometry (HPLC-MS) Analysis

All samples were acquired by the HPLC-MS system following machine instructions. The analytical conditions were as follows, UPLC: column, Waters ACQUITY UPLC HSS T3 1.8 µm, 2.1 mm × 100 mm (Waters Corporation, MA, USA); column temperature, 40 °C; flow rate, 0.40 mL/min; injection volume, 4 μL; solvent system, water (0.1% formic acid): acetonitrile (0.1% formic acid); Sample measurements were performed with a gradient program that employed the starting conditions of 95% A, 5% B. Within 5 min, a linear gradient to 35% A, 65% B was programmed; within 1 min, a linear gradient to 1% A, 99% B was programmed, and maintained for 1.5 min. Subsequently, a composition of 95% A, 5.0% B was adjusted within 0.1 min and maintained for 2.4 min.

Data were acquired using the information-dependent acquisition (IDA) mode using Analyst TF 1.7.1 Software (Sciex, Concord, ON, Canada). The source parameters were set as follows: ion source gas 1 (GAS1), 50 psi; ion source gas 2 (GAS2), 60 psi; curtain gas (CUR), 35 psi; temperature (TEM), 550 °C or 550 °C; declustering potential (DP), 80 V or −80 V in positive or negative modes, respectively; and ion spray voltage floating (ISVF), 5500 V or −4500 V in positive or negative modes, respectively. The TOF MS scan parameters were set as follows: mass range, 50–1250 Da; accumulation time, 200 ms; and dynamic background subtract, on. The product ion scan parameters were set as follows: mass range, 50–1250 Da; accumulation time, 40 ms; collision energy, 30 or −30 V in positive or negative modes, respectively; collision energy spread, 15; resolution, UNIT; charge state, 1 to 1; intensity, 100 cps; exclude isotopes within 4 Da; mass tolerance, 50 mDa; maximum number of candidate ions to monitor per cycle, 12.

### 2.3. Data Processing and Annotation

Downstream data analysis of metabolomics was carried out using R (version 4.3.3) and relevant packages, including several key steps. Firstly, unsupervised principal component analysis (PCA) was conducted using the prcomp function in R, and the data were unit variance scaled before analysis. Secondly, hierarchical cluster analysis (HCA) and Pearson correlation coefficients (PCC) were calculated. HCA results for samples and metabolites were presented as heatmaps with dendrograms, while PCC between samples were calculated by the cor function in R and presented as heatmaps. Both HCA and PCC were implemented using the R package ComplexHeatmap, and the normalized signal intensities of metabolites (through unit variance scaling) in HCA were visualized as a color spectrum. For differential metabolite selection in two-group analysis, differential metabolites were determined by VIP (VIP > 1) and absolute Log_2_FC (|Log_2_FC| ≥ 1.0). The VIP values were extracted from the OPLS-DA results and were generated using the R package MetaboAnalystR. The data were log-transformed (log2) and mean-centered before OPLS-DA, and a permutation test (200 permutations) was performed to avoid overfitting. Finally, the identified metabolites were annotated using the KEGG Compound database (http://www.kegg.jp/kegg/compound/, accessed on 1 January 2025, Release 113.0), and the annotated metabolites were then mapped to the KEGG Pathway database (http://www.kegg.jp/kegg/pathway.html, accessed on 1 January 2025, Release 113.0). In addition, TBtools v2.225 and the Majorbio Cloud Platform (https://cloud.metware.cn/, accessed on 20 April 2025) were also used for heatmap construction and KEGG enrichment analysis.

## 3. Results and Discussion

### 3.1. Metabolome Data Evaluation

**Figure 1:**
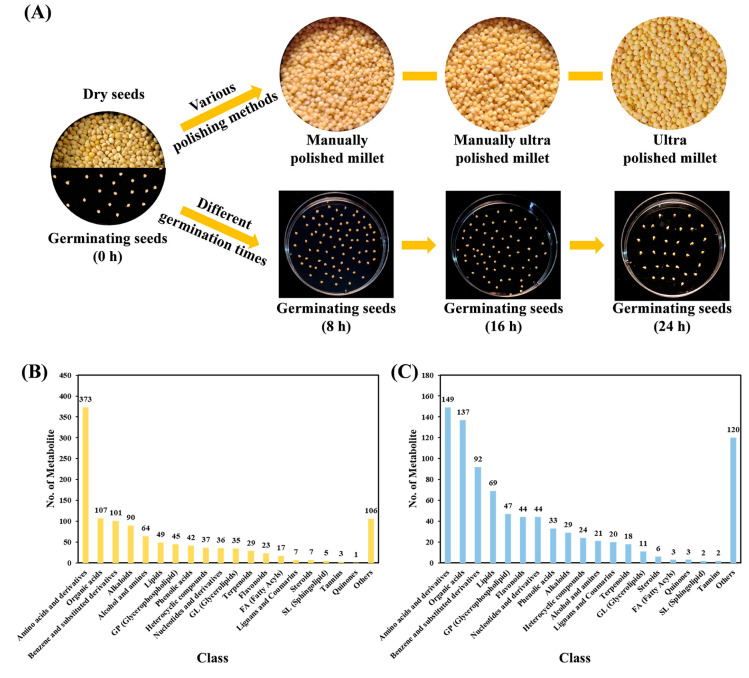
Experimental design and detected metabolites in this study. (**A**) Experimental samples for metabolome detection; (**B**,**C**) represent the number and classification of metabolites detected under the POS and NEG modes, respectively.

A non-targeted metabolomics technique was used to detect the metabolites of millet and seeds germinated for 8 h, 16 h, and 24 h under different processing methods (Millet: DS, MPM, UPM and MUPM; Germinating seeds: FDS and ODS) (Figure 1A). The quality control sample (QC, with three repetitions) composed of a mixture of all 30 samples was used to analyze the inter-sample repeatability. The results of the overlapping analysis of the total ion current (TIC) chromatograms of the QC samples showed that the curves of the total ion current had a high degree of overlap under both positive (Pos) and negative (Neg) scanning modes, and the retention times and peak intensities were consistent, confirming the good stability of data collection and the reliability of the results (Figure S1A,B). The distribution diagram of the coefficient of variation (CV) values of the samples showed that, under both modes, the proportion of metabolites with a CV value less than 0.5 in all samples was higher than 75%, and the proportion of metabolites with a CV value less than 0.3 was higher than 50%. The proportion of metabolites with a CV value less than 0.3 in the QC samples was higher than 85%, indicating that the experimental data were highly stable (Figure S1C,D). Meanwhile, the correlation of the QC samples was all above 0.99 (Figure S1E,F). All the above quality-control results confirmed that the metabolomic data of the 30 samples obtained by us were of high quality and could be used for downstream analysis.

### 3.2. Metabolite Composition Analysis

Based on the high-performance liquid chromatography-mass spectrometry (HPLC-MS) technique, a total of 2054 metabolites were detected in 30 samples. In the positive ion mode and negative ion mode, 1180 and 874 metabolites were detected, respectively (Figure 1B,C). These metabolites were classified into 20 categories, with major classes including Amino acids and derivatives (522, 25.45%), Organic acids (244, 11.90%), Benzene and substituted derivatives (193, 9.41%), Alkaloids (119, 5.80%), Lipids (118, 5.75%), Glycerophospholipid (GP, 92, 4.49%), Alcohol and amines (85, 4.14%), Nucleotides and derivatives (80, 3.90%), Phenolic acids (75, 3.66%), and Flavonoids (67, 3.27%). Metabolite compositions were similar across samples, but their abundances differed significantly.

Amino acids and derivatives, primarily derived from endosperm storage protein degradation, provide nitrogen sources for de novo tissue synthesis. Organic acids, as intermediates of glycolysis and the tricarboxylic acid (TCA) cycle, reflect carbon metabolism and energy status. Dynamic changes in lipids (including glycerolipids and glycerophospholipids) are directly associated with storage lipid hydrolysis and membrane lipid remodeling, with degradation products contributing to gluconeogenesis and cell membrane assembly. Phenylpropanoid derivatives (e.g., phenolic acids and flavonoids), generated via the phenylalanine metabolic pathway, serve as precursors for cell wall components (e.g., lignin) and exhibit secondary metabolic functions such as antioxidant activity. Nucleotides and derivatives, derived from nucleic acid degradation and de novo synthesis, provide substrates for DNA replication and energy transfer (e.g., ATP) during germination [36]. These differential accumulations of metabolites are highly coordinated with the material demands of starch/protein degradation and embryo development during seed germination. Similar metabolic profiling trends have been observed in germinating cereals like rice [37] and wheat [35], confirming the conserved functional consistency of metabolic networks across grass species during germination.

### 3.3. Principal Component Analysis (PCA) and Partial Least Squares-Discriminant Analysis (PLS-DA)

**Figure 2:**
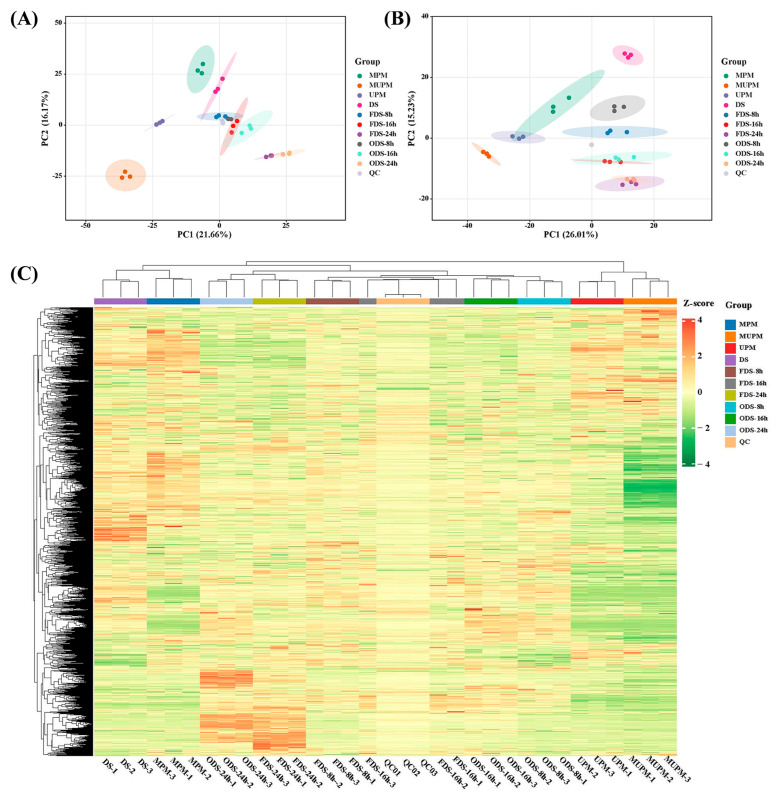
PCA and cluster analysis of metabolites in all samples. (**A**,**B**) represent PCA plots in POS and NEG modes, respectively, (**C**) Heatmap of metabolites content detected in all samples, with colors from green to red indicating increasing metabolite content.

To characterize inter- and intra-group metabolic variations, unsupervised PCA and supervised PLS-DA were performed on all samples based on metabolite abundances [39]. PCA results showed clear separation of most sample groups under both POS and NEG modes (Figure 2A,B). The three polished millet groups (MPM, MUPM, UPM) were completely distinct from the dry seed (DS) group, indicating substantial metabolic alterations induced by different polishing methods. During germination, samples at 8 h, 16 h, and 24 h were clearly separated, while subgroups with different processing methods (FDS vs. ODS) at the same germination time clustered closely, suggesting that germination-driven metabolic changes far exceeded the effects of processing [35,36].

PLS-DA, a supervised multivariate statistical method, maximizes inter-group discrimination by extracting correlated components between independent variables (X) and dependent variables (Y), facilitating the identification of differential metabolites. PLS-DA results showed complete separation of all sample groups, with high intra-group clustering in both QC and sample groups, indicating minimal biological replicate variation (Figure S2A,B). In both ion modes, R^2^ values exceeded Q^2^ values (Figure S2C,D), confirming no overfitting and validating the method for discovering inter-group differential metabolites. Hierarchical clustering of metabolites revealed that DS and MPM clustered together, while UPM and MUPM formed a distinct cluster (Figure 2C). Metabolites at 8 h and 16 h germination were more similar to each other than to those at 24 h, highlighting significant metabolic shifts across germination stages and processing methods—especially in UPM, MUPM, FDS-24 h, and ODS-24 h groups.

### 3.4. Differential Metabolite Analysis of Millet Under Different Polishing Methods

**Figure 3:**
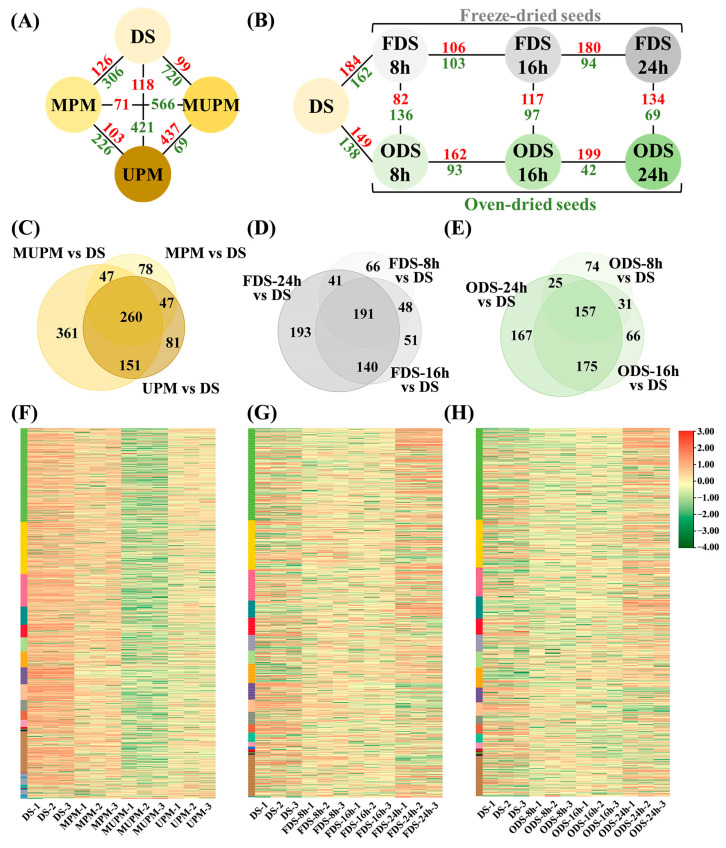
Differential metabolite analysis of millet and sprouted seeds under different polishing methods. (**A**,**B**) represent the differential metabolites of millet and sprouted seeds, respectively, with red representing upregulation and green representing downregulation. (**C**–**E**) represent common/unique differential metabolites between different groups. (**F**–**H**) show the contents of differential metabolites corresponding to (**C**–**E**).

To investigate the effects of polishing methods on millet metabolites, differential metabolites were screened using Variable Importance in Projection (VIP > 1) from Orthogonal Partial Least Squares-Discriminant Analysis (OPLS-DA) combined with |Log_2_(Fold Change)| ≥ 1. Compared to whole grains (DS), all three polishing methods significantly downregulated numerous metabolites: 306, 421, and 720 metabolites in MPM, UPM, and MUPM, respectively, correlating with their dehulling intensities (Figure 3A). The most aggressive polishing method MUPM exhibited the most severe metabolite loss, showing 566 and 437 more downregulated metabolites than MPM and UPM, respectively. The drastic reduction in metabolite abundances in MUPM was evident across all categories (Figure 3F).

**Figure 4:**
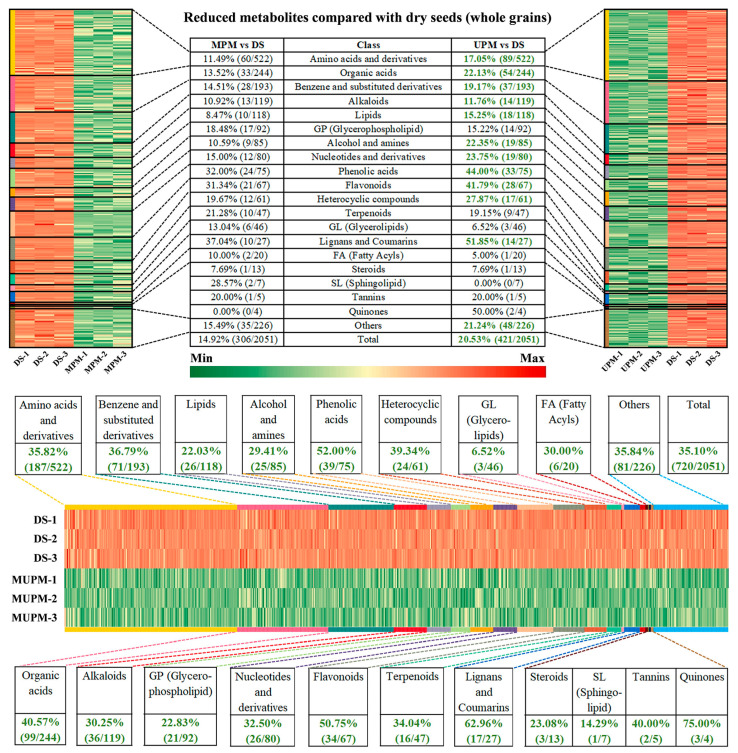
Metabolite changes in millets under different polishing methods. The downregulation ratios and quantities of different types of metabolites are listed in the table, and the metabolite categories that are consistently downregulated are marked in green font.

Specifically, MPM contained 306 downregulated metabolites (14.92% of total identified), classified into 19 groups, including Amino acids and derivatives (11.49%, 60/522), Organic acids (13.52%, 33/244), Benzene and substituted derivatives (14.51%, 28/193), Alkaloids (10.92%, 13/119), Lipids (8.47%, 10/118), Glycerophospholipid (18.48%, 17/92), Phenolic acids (32.00%, 24/75), and Flavonoids (31.34%, 21/67) (Figure 4). UPM showed 421 downregulated metabolites (20.53% of total), with broader decreases in categories like amino acids, organic acids, and phenolic compounds attributed to the removal of the seed coat, which is rich in antioxidants [38,40]. Ultra polishing further stripped the aleurone layer and embryo, reducing amino acids (e.g., GABA), lipids, and nucleotides stored in these tissues [41,42].

Intensive polishing, or MUPM, exhibited the most severe metabolic depletion, with 35.10% (720/2051) of metabolites significantly downregulated compared to DS (Figure 4). Except for glycerolipids and sphingolipids, all 20 categories showed >20% decreases, with Phenolic acids, Flavonoids, Lignans and Coumarins, and Quinones declining by over 50%. Loss of these antioxidants reduces radical scavenging capacity and diminishes chronic disease prevention benefits [43]. Excessive dehulling in MUPM also exacerbated losses of Lipids, Glycerophospholipid, and Fatty Acyls, compromising nutritional quality [44].

In summary, millet polishing removes husk, seed coat, embryo, and aleurone layer, tissues rich in antioxidants (phenolic acids, flavonoids, lignans), functional amino acids (e.g., GABA), essential fatty acids, and vitamins, leading to diminished nutritional and health benefits. While polishing improves appearance and texture, it sacrifices natural bioactive components. Preserving whole grain structures (e.g., husk and embryo) is critical for maximizing millet nutrition, as excessive polishing results in “refined millet and nutritional hollowing”. Among the three methods, less dehulling such as MPM balanced nutrition and palatability, offering a product with both sensory appeal and higher nutritional value.

### 3.5. Differential Metabolite Analysis of Germinating Seeds at Different Stages

To clarify metabolic changes during seed germination and guide post-processing of germinated seeds, we analyzed metabolites in seeds germinated for 8 h, 16 h, and 24 h under FD and HAD conditions (Figure 1A). Overall, germination induced significant metabolic changes, with the number of upregulated metabolites significantly exceeding downregulated ones, increasing progressively with germination time and peaking at 24 h (Figure 3B). A subset of metabolites showed continuous changes throughout germination: 191 and 157 metabolites exhibited consistent differential accumulation in FDS and ODS, respectively (Figure 3D,E). By contrast, stage-specific up/downregulated metabolites were relatively rare, especially at 8 h and 16 h. Compared to DS, FDS-8 h, FDS-16 h, ODS-8 h, and ODS-16 h had 66, 51, 74, and 66 stage-specific differentially accumulated metabolites, respectively, while FDS-24 h and ODS-24 h had 193 and 167, highlighting 24 h as a critical time point with distinct metabolic profiles compared to early stages (Figure 3G,H). These results indicate that seed germination is a coherent, dynamic process driven by core differentially accumulated metabolites, providing metabolic insights for stage-specific post-processing of germinated grain.

**Figure 5:**
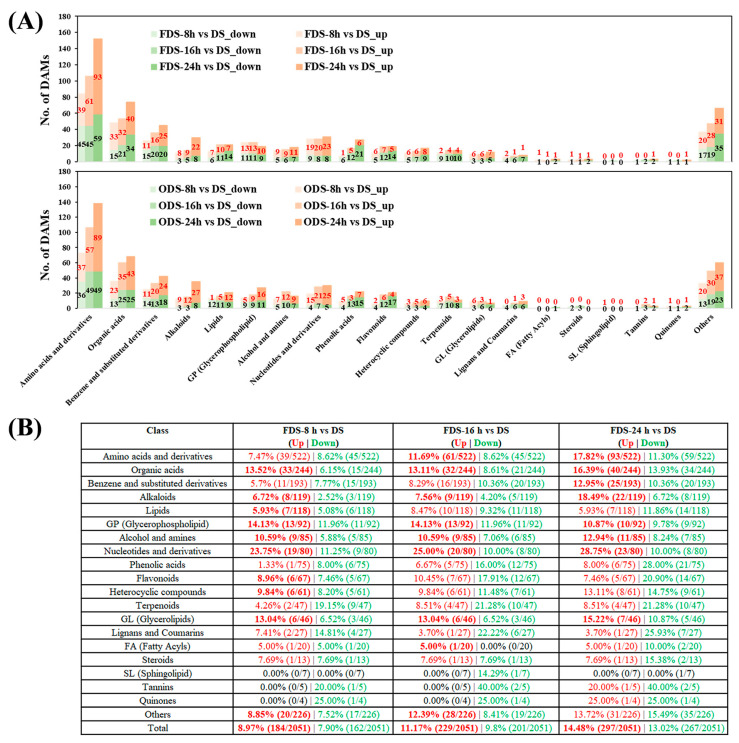
Metabolite changes in sprouted seeds at different germination stages. (**A**) Histograms of the numbers of differentially accumulated metabolites (DAMs) in seeds at different germination stages. (**B**) The upregulation and downregulation ratios and quantities of different types of metabolites in FDSs. Metabolite categories with a greater number of upregulated metabolites than downregulated ones are shown in bold.

In terms of metabolite categories, nearly all key classes showed significant changes during germination, reflecting comprehensive activation of metabolic pathways (Figure 5A). The number of differentially accumulated metabolites increased with germination time: at 8 h, 184 metabolites (8.97% of total) were upregulated vs. 162 (7.9%) downregulated; at 16 h, upregulated metabolites rose to 229 (11.17%) vs. 201 (9.8%) downregulated; at 24 h, upregulated metabolites peaked at 297 (14.48%) vs. 267 (13.02%) downregulated (Figure 5B). A similar trend was observed in ODS samples, with upregulated metabolites accounting for 7.26%, 11.02%, and 15.02% from 8 h to 24 h, while downregulated metabolites peaked at 10.53% at 24 h (Figure S3). These data indicate that germination preferentially activates metabolic pathways to meet energy and material demands, consistent with reports in other crops [45].

Specifically, Amino acids and derivatives, Nucleotides and derivatives, Organic acids, Alkaloids, and Alcohol and amines were continuously upregulated from 8 h to 24 h (Figure 5B). Upregulation of amino acids and nucleotides supports genetic information transfer, protein synthesis, and cell division, enhancing nutritional quality and bioavailability [27]. Organic acids fuel respiration and energy metabolism [46], while alcohol and amines modulate cellular responses to environmental cues, promoting seedling growth. Conversely, Phenolic acids, Flavonoids, Terpenoids, Lignans, and Coumarins had more downregulated than upregulated metabolites, particularly at 24 h, indicating reduced defensive investments—potentially compromising antioxidant and antibacterial properties, as well as anti-inflammatory nutritional quality (Figure 5B).

Thus, according to different nutritional needs, it is necessary to select seeds at appropriate germination stages for post-processing: early-stage germinated seeds retain more basic nutrients and anti-inflammatory/antioxidant compounds, while late-stage germinated seeds generate more other bioactive components.

### 3.6. KEGG Enrichment Analysis of Differential Metabolites in Millet Under Different Polishing Methods

**Figure 6:**
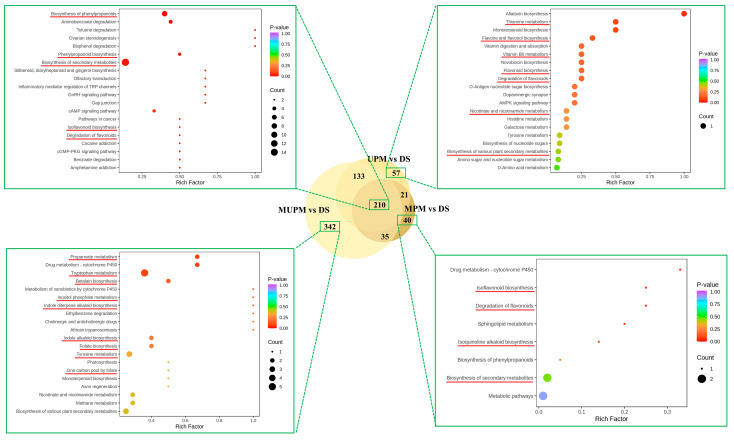
KEGG enrichment analysis of differential metabolites in millets under different polishing methods. The important KEGG pathways are marked with red underline.

To clarify the metabolic pathways in which the lost metabolites in millets with different polishing methods are involved, we conducted KEGG enrichment analysis. Compared with DS, a total of 210 metabolites were downregulated in the three types of polished millet, while UPM, MPM, and MUPM had 57, 40, and 342 specifically downregulated metabolites, respectively (Figure 6). The 210 commonly differential metabolites were mainly enriched in metabolic pathways such as Biosynthesis of phenylpropanoids, Biosynthesis of secondary metabolites, Isoflavonoid biosynthesis, and Degradation of flavonoids. These pathways are closely related to the synthesis of many important functional components in millet, such as lignin, coumarin, and flavonoids, which possess antioxidant, anti-inflammatory, and free-radical scavenging properties. The downregulation of these pathways directly reduces the antioxidant capacity and free-radical scavenging ability of millet, as well as its potential health benefits such as anti-inflammation properties, regulation of gut microbiota, anti-cancer properties, and glucose regulation [10,11,12,17,18]. For UPM, in addition to being enriched in Flavone and flavonol biosynthesis, Flavonoid biosynthesis, Degradation of flavonoids, and Biosynthesis of secondary metabolites, which directly weaken the antioxidant activity and health benefits of millet, its downregulated metabolites were also enriched in vitamin-related metabolic pathways (thiamine metabolism, vitamin B6 metabolism, nicotinate, and nicotinamide metabolism), as well as nucleotide sugar and amino sugar metabolism (Biosynthesis of nucleotide sugars, Amino sugar and nucleotide sugar metabolism). The downregulation of vitamin metabolic pathways led to a decrease in the content of vitamins B1, B6, and nicotinic acid in millet, reducing its value as a “nutrient-fortified cereal” [47]. The downregulation of nucleotide sugar and amino sugar metabolic pathways may cause structural changes in dietary fiber (such as β-glucan and arabinoxylan), affecting the slow-digesting starch characteristics and prebiotic function of millet in the intestine [48]. The downregulated metabolites in MPM were similar to those in UPM, mainly enriched in antioxidant-functional-component-related synthesis pathways such as Isoquinoline alkaloid biosynthesis, Isoflavonoid biosynthesis, and Degradation of flavonoids.

MUPM contained the largest number of 342 specifically downregulated metabolites, which were mainly enriched in Propanoate metabolism, Tryptophan metabolism, Tyrosine metabolism, Betalain biosynthesis, Inositol phosphate metabolism, Indole diterpene alkaloid biosynthesis, Folate biosynthesis, One carbon pool by folate, and Monoterpenoid biosynthesis pathways. The downregulation of these pathways all directly affected the nutritional quality of germinated seeds. The downregulation of the propionate metabolism pathway affected the short-chain fatty acid precursors in millet, reducing its functions of regulating the intestinal barrier and enhancing immunity [48,49]. The downregulation of tryptophan, tyrosine metabolism pathways, and folate synthesis-related metabolic pathways directly reduced the basic nutritional value of millet [8,50]. The downregulation of the Monoterpenoid biosynthesis pathway led to a decrease in monoterpenoids, directly reducing the characteristic aroma and flavor complexity of millet. The downregulation of indole alkaloid and betaine metabolism pathways directly led to a decrease in the content of these secondary metabolites and antioxidants in millet, weakening its anti-inflammatory, antioxidant, and other health benefits [15,18].

Overall, as the mechanical polishing intensity increased from the weakest MPM to UPM and then to MUPM, the bran, seed coat, embryo and other parts were removed, directly resulting in a comprehensive loss of basic nutrients (essential amino acids, vitamins, dietary fiber, etc.), functional active components (indole alkaloids, betaine, phenolic acids, flavonoids), and flavor substances (such as monoterpenoids) in millet, causing serious damage to its nutritional value. Therefore, during the polishing, it is necessary to balance the degree of dehulling as much as possible and retain part of the bran or embryo to maximize the retention of nutrients and functional components, especially for millet products that emphasize health value. Further investigation may be carried out to dissect the changes in the kernel structure after different polishing methods and determine the definite levels of metabolites using targeted metabolics, to reveal how much of the functional nutrients is lost due to intensive polishing, providing a stonger reference for millet processing factories and consumers.

### 3.7. KEGG Enrichment Analysis of Differential Metabolites in Germinating Seeds at Different Stages

**Figure 7:**
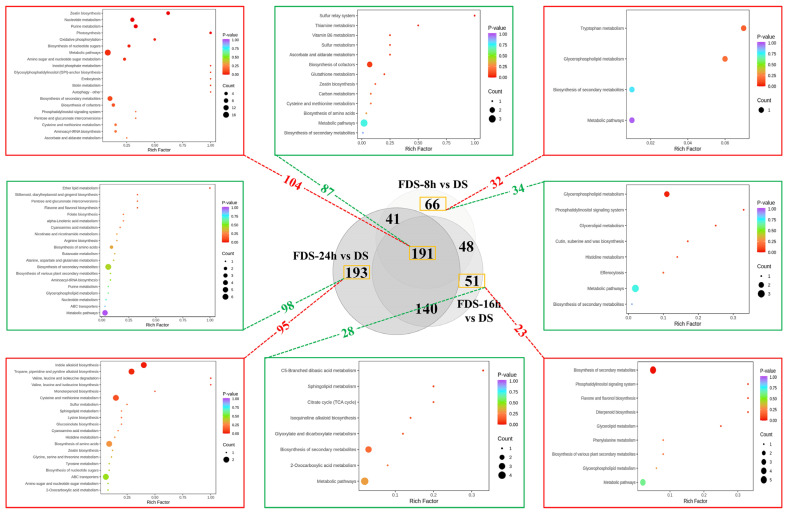
KEGG enrichment analysis of differential metabolites in sprouted seeds at different germination stages. The number of up-regulated and down-regulated metabolites is indicated by red and green fonts, respectively.

As described above, significant metabolite changes occur during seed germination, including 191 metabolites that change steadily throughout the process, with 104 upregulated and 87 downregulated (Figure 7). These common differential metabolites have a continuous impact on foxtail millet germination and nutrient accumulation. KEGG analysis results show that upregulated metabolites are significantly enriched in multiple energy and basic metabolic pathways (such as Oxidative phosphorylation, Metabolic pathways), nucleic acid and cell division-related pathways (Nucleotide metabolism, Zeatin biosynthesis), cell wall and storage substance conversion pathways (such as Amino sugar and nucleotide sugar metabolism), antioxidant and secondary metabolic pathways (Biosynthesis of secondary metabolites, Cysteine and methionine metabolism), and vitamin and coenzyme synthesis pathways (Biosynthesis of cofactors, Ascorbate and aldarate metabolism).

These pathways are closely related to energy and material mobilization, cell division and growth, and antioxidant stress during seed germination. They also promote the nutritional enhancement of seeds. For example, through metabolic reprogramming, starch is degraded into small molecule sugars such as glucose and maltose, increasing absorbability (Oxidative phosphorylation); protein decomposition and de novo synthesis release essential amino acids, promoting amino acid balance and enhancing protein nutritional value (Cysteine and methionine metabolism) [23,24]; antioxidant components such as vitamin B, vitamin C, and flavonoids are enriched (Biosynthesis of secondary metabolites) [25]; bioactive substances such as nucleotides, coenzymes, zeatin, and cell wall polysaccharides are accumulated, promoting their roles in intestinal health, immune regulation, etc. (Nucleotide metabolism, Zeatin biosynthesis, Biosynthesis of nucleotide sugars, Amino sugar and nucleotide sugar metabolism) [22,28].

On the other hand, the KEGG enrichment results of downregulated metabolites also confirm the degradation of storage substances, the release of basic nutrients, and the reconstruction of metabolic pathways and nutritional enhancement during germination. The downregulation of starch and protein synthesis pathways such as Carbon metabolism and Biosynthesis of amino acids releases basic nutrients such as glucose and essential amino acids, increasing the protein nutrients and absorbability of germinated seeds; the downregulation of pathways such as Biosynthesis of cofactors, Thiamine metabolism, and Vitamin B6 metabolism increases the active forms of B-group vitamins (thiamine, B6) and vitamin C. These changes in differential metabolites during seed germination directly improve the digestibility, essential nutrients (amino acids, vitamins), antioxidant components, and bioactive substances of germinated seeds, making them a food raw material with higher nutritional density and stronger functionality.

Regarding different stages of seed germination, there are relatively few specific differential metabolites in the early germination stages of 8 h and 16 h, with 66 (32 upregulated and 34 downregulated) and 51 (23 upregulated and 28 downregulated), respectively. The 8 h specific differential metabolites are mainly enriched in pathways such as glycerophospholipid metabolism, glyceride metabolism, phosphatidylinositol signaling system, tryptophan metabolism, and biosynthesis of cuticle, suberin, and wax. These pathways lead to the accumulation of essential amino acids (such as tryptophan), fatty acids, and phospholipids, while reducing the wax components in the glume, enhancing basic nutrients and reducing the crude fiber content of seeds, thus improving seed nutrient content and digestibility [28]. The specific differential metabolites in 16 h germinated seeds are mainly enriched in pathways such as phenylalanine metabolism, diterpene/flavonoid synthesis, TCA cycle, and glyoxylate/dicarboxylate metabolism. At this stage, secondary metabolites begin to accumulate in large quantities, and the antioxidant capacity is enhanced. Meanwhile, the downregulation of the TCA cycle indicates a shift in energy metabolism from “complete glucose oxidation” to “intermediate product synthesis” at this stage, promoting the accumulation of organic acids and improving the taste. At the late germination stage of 24 h, a large number of specific differential metabolites appear in the seeds, with 95 upregulated and 98 downregulated. The upregulated metabolites are mainly enriched in nitrogen metabolism and special amino acid pathways (tyrosine/tryptophan metabolism, indole alkaloid synthesis, cysteine/methionine metabolism, sulfur metabolism), and energy and lipid metabolism reorganization (α-linolenic acid metabolism, sphingolipid metabolism). The downregulated metabolites are enriched in basic substance synthesis and energy metabolism related pathways (glycerophospholipid metabolism), and secondary metabolism and stress resistance related pathways (flavonoid/flavonol biosynthesis, dibenzyl/gingerol synthesis, cyanate metabolism, glucosinolate synthesis). These changes in metabolic pathways suggest that by 24 h, germinated seeds have shifted from the synthesis peak at 16 h to a process of functional specialization. The accumulation of functional lipids, indole containing compounds, and sulfur containing active components plays an important role in the efficacy of processed germinated-seed products in improving the intestine and preventing chronic diseases [18,51].

In general, germinated seeds at different stages are suitable for different nutritional processing needs. Early germinated (8 h) seeds produce more free amino acids, small-molecule sugars, essential fatty acids, and low anti-nutritional factors. In addition, the accumulation of γ-aminobutyric acid is highest at this stage, which may be more suitable for processing into easily digestible complementary foods for infants and the elderly, and also has certain effects on blood pressure, blood lipid, and blood sugar control, as well as enhancing memory (Table S1) [43]. The 16 h germination stage is important for the accumulation of secondary antioxidant metabolites such as flavonoids, and germinated seeds at this stage are more suitable for the development of highly active, functional health products. The 24 h germination seed specializes in sulfur-containing metabolites and indole-containing functional components, and seems more suitable for the development of “precision nutrition-targeted products”.

### 3.8. KEGG Enrichment Analysis of Differential Metabolites in Germinating Seeds with Different Processing Methods

**Figure 8:**
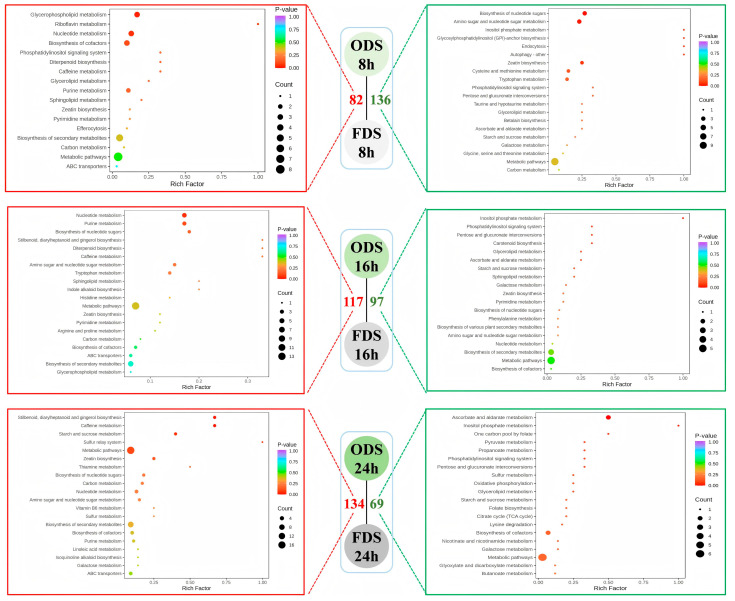
KEGG enrichment analysis of differential metabolites in sprouted seeds with Different Processing Methods. The number of up-regulated and down-regulated metabolites is indicated by red and green fonts, respectively.

Due to the different basic processes of the two drying methods, to compare the effects of different processing methods on the nutritional components of germinated seeds, we compared the metabolite differences between hot air drying and freeze drying at each germination stage. Hot air drying rapidly removes moisture through thermal effects, while FD dehydrates at low temperatures to maximally preserve heat sensitive components. Their impacts on nutrients showed significant differences according to the metabolic characteristics at different germination stages (8 h/16 h/24 h) (Figure 8).

Specifically, at the initial germination stage (8 h), compared with FDS-8 h, the upregulated metabolites in ODS-8 h were mainly enriched in metabolic pathways such as Glycerophospholipid metabolism, Sphingolipid metabolism, Biosynthesis of secondary metabolites, and Purine/Pyrimidine metabolism & Nucleotide metabolism. The downregulated metabolites were mainly enriched in Amino sugar and nucleotide sugar metabolism, Starch and sucrose metabolism, Cysteine and methionine metabolism, and Glycine, serine and threonine metabolism, HAD activated membrane lipid metabolism, nucleotide synthesis, and secondary metabolism, increasing functional components (phospholipids, antioxidants, nucleotides). However, it inhibited sulfur-containing amino acid and vitamin C synthesis and starch degradation, resulting in a decrease in essential amino acid and soluble sugar content. FD, on the other hand, had the advantage of preserving starch, sucrose metabolism, and cell wall synthesis, maintaining high levels of dietary fiber, vitamin C, and essential amino acids.

At the middle germination stage (16 h), HAD upregulated tryptophan metabolism, indole alkaloid synthesis, and sphingolipid/glycerophospholipid metabolism pathways, while downregulating phenylalanine metabolism and carotenoid synthesis, which may reduce the synthesis of vitamin A precursors (β-carotene) and aromatic amino acids (phenylalanine), FD-retained carbohydrates (starch and sucrose), and cell wall metabolism, maintaining more complete basic nutrients (carbohydrates and dietary fiber). At the late germination stage (24 h), HAD upregulated tyrosine metabolism and secondary metabolism, potentially accumulating more polyphenols, while downregulating the TCA cycle and oxidative phosphorylation (hindering energy metabolism), leading to reduced ATP production and excessive consumption of storage substances (starch and fat), and a decrease in the total amount of nutrients. FD maintained the efficiency of the TCA cycle and carbon metabolism, reducing excessive degradation of storage substances and retaining more original nutrients (such as starch and fatty acids).

Overall, FDS maintained a more complete basic metabolism (carbohydrates, amino acids, vitamins) at all time points, avoiding the inhibition of enzyme activity and material degradation (such as vitamin C destruction and excessive starch consumption) caused by high temperatures in ODS. However, ODS was suitable for preparing basic food ingredients with low anti-nutritional factors (such as infant rice flour), using high temperatures to degrade waxes and some alkaloids, especially for processing at the early germination stage (8 h).

### 3.9. Representative Metabolites Affected by Different Processing Methods and Germination Times

**Figure 9:**
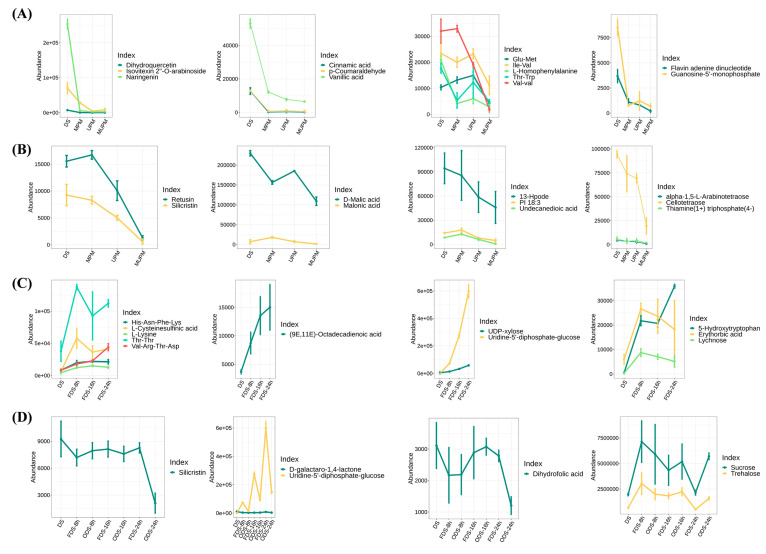
Changes in the content of representative metabolites in millet and germinating seeds under different processing methods. (**A**,**B**) represent the metabolic changes in millet polished under different methods, while (**C**,**D**) represent the metabolic changes in germinated seeds after drying and freeze-drying treatments.

In addition to analyzing metabolite changes under different processing methods and germination times based on metabolite categories and their pathways, we examined the changes in important metabolites in foxtail millet seeds. Compared with whole grains, the three different polishing methods led to a comprehensive downregulation of various flavonoids, phenolic acids, oligopeptides, and nucleotides (Figure 9A). Flavonoid (C-glycoside) compounds such as Dihydroquercetin, Naringenin, and Isovitexin 2″-O-arabinoside are the main contributors to the antioxidant and anti-inflammatory properties of millets, and their presence is closely related to the nutritional and health care functions of millets [52,53,54]. Phenolic acids represented by Cinnamic acid, the main antioxidant components in millet bran, can inhibit oxidative stress and improve intestinal health [53,55]. A large number of oligopeptides, vitamin B2 derivatives, and nucleotides (GMP) were also continuously downregulated with the increase in polishing intensity, which seriously affected the nutritional quality of the millets (Figure 9A). Notably, ultra-deep polishing (MUPM) also downregulated the contents of various organic acids such as D-Malic acid and Malonic acid, free fatty acids such as 13-Hpode and Undecanedioic acid, and essential amino acids such as L-Tryptophan and DL-Arginine, further affecting the nutritional quality of millets (Figure 9B, Table S1).

During seed germination, a large number of nutrients were significantly increased. Lysine, one of the most deficient essential amino acids in the edible parts of gramineous crops, was significantly enhanced in germinated seeds (Figure 9C). The well-known γ-aminobutyric acid was also significantly enhanced in 8 h germinated seeds (Table S1). In addition, various functional components such as 5-Hydroxytryptophan, (9E,11E)-Octadecadienoic acid, and oligopeptides were found to continuously accumulate during germination (Figure 9C). It has been reported that 5-Hydroxytryptophan plays an important role in regulating human mood, sleep, appetite, body temperature, and pain perception [56]. Moreover, we found a significant downregulation of Dihydrofolic acid and Silicristin under hot air drying, especially at 24 h of germination (Figure 9D). As a precursor of folic acid, Dihydrofolic acid was significantly downregulated by hot air drying, directly reducing the B-vitamin nutrition in germinated seeds. Silicristin is an important antioxidant and anti-inflammatory component that protects the cardiovascular system and inhibits oxidative stress. The high temperature of hot-air drying significantly reduced its content, resulting in a significant decrease in the antioxidant activity of germinated seeds.

In conclusion, different polishing methods can lead to the downregulation of various nutrients and functional components such as flavonoids, phenolic acids, oligopeptides, and nucleotides in millet, with deep polishing having a more severe impact. Seed germination can significantly increase the content of essential amino acids such as lysine and functional components such as γ-aminobutyric acid and 5-hydroxytryptophan. However, hot air drying can reduce the content of some B-vitamin precursors and antioxidant components. Evidently, the effects of different processing methods and germination times on the nutritional quality of millet are complex, and it is necessary to comprehensively optimize processing and germination conditions to maximize the retention or improvement of its nutritional quality.

## 4. Conclusions

In this study, we employed comparative metabolomics to comprehensively explore the metabolite changes in millets and germinated seeds under different processing methods. Polishing methods significantly impact the nutritional quality of millets. MPM, UPM, and MUPM all led to the downregulation of numerous metabolites. The loss of antioxidants, essential amino acids, fatty acids, and vitamins was particularly notable, with the degree of reduction correlating with polishing intensity. MUPM in particular caused the most severe depletion of these nutrients, highlighting the importance of balanced polishing to retain nutritional value. Among the polishing methods investigated, MPM emerged as a more favorable option, offering a better balance between nutrition and palatability. Conversely, germination triggered metabolic pathways to accumulate essential nutrients and bioactive compounds with stage specificity: early stages (8 h) enriched free amino acids and small sugars; 16 h stages prioritized antioxidant secondary metabolites (e.g., flavonoids); and 24 h stages specialized in sulfur-containing functional components. These profiles support targeted product development, such as infant foods (early stages) or antioxidant supplements (mid-stages). Additionally, drying methods differed in nutrient retention: FD preserved basic nutrients (carbohydrates and vitamins), while HAD reduced heat-sensitive components but increased antioxidants, suitable for low anti-nutrient ingredients. In conclusion, our study provides a comprehensive framework for understanding the nutrient changes in millet and germinated seeds under different processing methods, highlighting the need for balanced processing (e.g., moderate dehulling and stage-specific germination) to maximize nutritional value. Future research could focus on mechanistic insights into metabolic reprogramming during germination and processing, enabling targeted strategies to enhance the nutritional and health benefits of foxtail millet.

## Data Availability

The original contributions presented in this study are included in the article/Supplementary Materials. Further inquiries can be directed to the corresponding author.

## Supplementary Materials

The following supporting information can be downloaded at: https://www.mdpi.com/article/10.3390/foods14111900/s1, Figure S1: Quality assessment of metabolome assays. (A) the total ion current (TIC) chromatograms of the QC samples under Pos scanning mode. (B) the total ion current (TIC) chromatograms of the QC samples under Neg scanning mode. (C) Distribution map of CV value of all samples under Pos scanning mode. (D) Distribution map of CV value of all samples under Neg scanning mode. (E) Correlation analysis of QC samples under Pos scanning mode. (F) Correlation analysis of QC samples under Neg scanning mode. Figure S2: Partial least squares discriminant analysis under Pos and Neg modes. (A) Partial least squares discriminant analysis under Pos mode. (B) Partial least squares discriminant analysis under Neg mode. (C) Permutation testing of the partial least squares discriminant analysis model in Pos mode. (D) Permutation testing of the partial least squares discriminant analysis model in Neg mode. Figure S3: The upregulation and downregulation ratios and quantities of different types of metabolites in ODSs. Metabolite categories with a greater number of upregulated metabolites than downregulated ones are shown in bold. Figure S4. Original KEGG enrichment analysis results of differential metabolites in millet under different polishing methods. (A) The KEGG enrichment analysis dot plot of 210 common differential metabolites in UPM (Ultra-Polished Millet), MUPM (Manually Ultra-Polished Millet) and MPM (Manually Polished Millet) upon comparison with DS (Dry Seed). (B) The KEGG enrichment analysis dot plot of 57 metabolites with specific differential accumulation in UPM. (C) The KEGG enrichment analysis dot plot of 342 metabolites with specific differential accumulation in MUPM. (D) The KEGG enrichment analysis dot plot of 40 metabolites with specific differential accumulation in MPM. Figure S5. Original KEGG enrichment analysis results of differential metabolites in sprouted seeds at different germination stages. (A,B) represent dot plots of KEGG enrichment analysis for 104 upregulated and 87 downregulated metabolites in sprouted seeds at 8 h, 16 h, and 24 h compared to dry seeds, respectively. (C,D) represent dot plots of KEGG enrichment analysis for specifically 32 upregulated and 34 downregulated metabolites in germinating seeds at 8 h, respectively. (E,F) represent dot plots of KEGG enrichment analysis for specifically 28 upregulated and 23 downregulated metabolites in germinating seeds at 16 h, respectively. (G,H) represent dot plots of KEGG enrichment analysis for specifically 95 upregulated and 98 downregulated metabolites in germinating seeds at 24 h, respectively. Figure S6. Original KEGG enrichment analysis results of differential metabolites in sprouted seeds with different processing methods. (A,B) represent KEGG enrichment dot plots of 82 upregulated and 136 downregulated metabolites, respectively, in 8-h germinated seeds under freeze-drying and oven-drying treatments. (C,D) represent KEGG enrichment dot plots of 117 upregulated and 97 downregulated metabolites, respectively, in 16-h germinated seeds under freeze-drying and oven-drying treatments. (E,F) represent KEGG enrichment dot plots of 134 upregulated and 69 downregulated metabolites, respectively, in 24-h germinated seeds under freeze-drying and oven-drying treatments. Table S1. The basic information of all 2054 metabolites was detected in this study.

## Abbreviations

The following abbreviations are used in this manuscript:

MPM	Manually Polished Millet
UPM	Ultra-Polished Millet
MUPM	Manually Ultra-Polished Millet
HAD	Hot Air Drying
FD	Freeze-Drying
DS	Dry Seed
FDS	Freeze-Dried Seed
ODS	Overn-Dried Seed

## References

[1] Samtiya M., Aluko R.E., Dhaka N., Dhewa T., Puniya A.K. (2023). Nutritional and health-promoting attributes of millet: Current and future perspectives. Nutr. Rev..

[2] Li P., Cai X., Li S., Zhao W., Liu J., Zhang X., Zhang A., Guo L., Li Z., Liu J. (2024). Nutrient and metabolite characteristics of the husk, bran and millet isolated from the foxtail millet (*Setaria italica* L.) during polishing. Food Chem. X.

[3] Zhu Y., Chen C., Dai Z., Wang H., Zhang Y., Zhao Q., Xue Y., Shen Q. (2024). Identification, screening and molecular mechanisms of natural stable angiotensin-converting enzyme (ACE) inhibitory peptides from foxtail millet protein hydrolysates: A combined in silico and in vitro study. Food Funct..

[4] Wang H., Shen Q., Fu Y., Liu Z., Wu T., Wang C., Zhao Q. (2023). Effects on Diabetic Mice of Consuming Lipid Extracted from Foxtail Millet (*Setaria italica*): Gut Microbiota Analysis and Serum Metabolomics. J. Agric. Food Chem..

[5] Zhang W., Zhang G., Liang W., Tian J., Sun S., Zhang X., Lv X., Guo P., Qu A., Wu Z. (2024). Structure, Functional Properties, and Applications of Foxtail Millet Prolamin: A Review. Biomolecules.

[6] Farzana T., Abedin M.J., Abdullah A.T.M., Reaz A.H., Bhuiyan M.N.I., Afrin S., Satter M.A. (2024). Enhancing prebiotic, antioxidant, and nutritional qualities of noodles: A collaborative strategy with foxtail millet and green banana flour. PLoS ONE.

[7] Sachdev N., Goomer S., Singh L.R.K., Chowhan R.K. (2024). Preparation and nutritional characterisation of protein concentrate prepared from foxtail millet (*Setaria italica*). Food Sci. Technol. Int..

[8] Hou S., Men Y., Wei M., Zhang Y., Li H., Sun Z., Han Y. (2022). Total Protein Content, Amino Acid Composition and Eating-Quality Evaluation of Foxtail Millet (*Setaria italica* (L.) P. Beauv). Foods.

[9] Abedin M.J., Abdullah A.T.M., Satter M.A., Farzana T. (2022). Physical, functional, nutritional and antioxidant properties of foxtail millet in Bangladesh. Heliyon.

[10] Suma P.F., Urooj A. (2012). Antioxidant activity of extracts from foxtail millet (*Setaria italica*). J. Food Sci. Technol..

[11] Dong J.L., Wang L., Lü J., Zhu Y.Y., Shen R.L. (2019). Structural, antioxidant and adsorption properties of dietary fiber from foxtail millet (*Setaria italica*) bran. J. Sci. Food. Agric..

[12] Shi J., Shan S., Li H., Song G., Li Z. (2017). Anti-inflammatory effects of millet bran derived-bound polyphenols in LPS-induced HT-29 cell via ROS/miR-149/Akt/NF-κB signaling pathway. Oncotarget.

[13] Pang M., He S., Wang L., Cao X., Cao L., Jiang S. (2014). Physicochemical properties, antioxidant activities and protective effect against acute ethanol-induced hepatic injury in mice of foxtail millet (*Setaria italica*) bran oil. Food Funct..

[14] Lu Y., Shan S., Li H., Shi J., Zhang X., Li Z. (2018). Reversal Effects of Bound Polyphenol from Foxtail Millet Bran on Multidrug Resistance in Human HCT-8/Fu Colorectal Cancer Cell. J. Agric. Food Chem..

[15] Shan S., Shi J., Li Z., Gao H., Shi T., Li Z., Li Z. (2015). Targeted anti-colon cancer activities of a millet bran-derived peroxidase were mediated by elevated ROS generation. Food Funct..

[16] Li Q., Wang X., Ma C., Onyango S., Wu W., Gao H., Li Q. (2025). Foxtail millet bran dietary fibres foster in vitro beneficial gut microbes and metabolites while suppressing pathobionts. Food Chem..

[17] Shan S., Lu Y., Zhang X., Shi J., Li H., Li Z. (2021). Inhibitory effect of bound polyphenol from foxtail millet bran on miR-149 methylation increases the chemosensitivity of human colorectal cancer HCT-8/Fu cells. Mol. Cell. Biochem..

[18] Yuxuan A., Xiaoqin L., Songtao L., Jinmiao T., Xiaxia F., Kaili C., Lichao Z., Zhuoyu L. (2023). Polyphenols from whole millet grain (*Setaria italica*) alleviate glucose and lipid homeostasis in diet-induced obese mice by increasing endogenous GLP-1. J. Sci. Food. Agric..

[19] Yang R., Shan S., An N., Liu F., Cui K., Shi J., Li H., Li Z. (2022). Polyphenols from foxtail millet bran ameliorate DSS-induced colitis by remodeling gut microbiome. Front. Nutr..

[20] Srinivasan A., Ekambaram S.P., Perumal S.S., Aruldhas J., Erusappan T. (2020). Chemical characterization and immunostimulatory activity of phenolic acid bound arabinoxylans derived from foxtail and barnyard millets. J. Food Biochem..

[21] Fu D., Wu W., Mustafa G., Yang Y., Yang P. (2025). Molecular mechanisms of rice seed germination. New Crops.

[22] Xu L., Wang X., Li Q., Niu Y., Ding G., He J., Chen W., Tian D. (2024). Optimization of γ-aminobutyric acid production in brown rice via prolonged seed priming. Plants.

[23] Barakat H., Al-Qabba M.M., Algonaiman R., Radhi K.S., Almutairi A.S., Al Zhrani M.M., Mohamed A. (2024). Impact of sprouting process on the protein quality of yellow and red quinoa (*Chenopodium quinoa*). Molecules.

[24] Bera I., O’Sullivan M., Flynn D., Shields D.C. (2023). Relationship between protein digestibility and the proteolysis of legume proteins during seed germination. Molecules.

[25] Avezum L., Madode Y.E., Mestres C., Achir N., Delpech C., Chapron M., Gibert O., Rajjou L., Rondet E. (2024). New insights into the rapid germination process of lentil and cowpea seeds: High thiamine and folate, and low α-galactoside content. Food Chem..

[26] Baranzelli J., Somacal S., Araujo Amorim Bonini C., Smaniotto F.A., Sant’Anna Monteiro C., Trivisiol da Silva D., de Oliveira Mello R., Ramos Boldori J., Casagrande Denardin C., Rodrigues E. (2023). Influence of sprouting on the bioaccessibility and bioactivity of benzoxazinoids, phenolic acids, and flavonoids of soft and hard wheat cultivars. Food Res. Int..

[27] Keawkim K., Lorjaroenphon Y., Vangnai K., Jom K.N. (2021). Metabolite–flavor profile, phenolic content, and antioxidant activity changes in sacha inchi (*Plukenetia volubilis* L.) seeds during germination. Foods.

[28] Maleki S., Razavi S.H., Yadav H. (2023). Diabetes and seeds: New horizon to promote human nutrition and anti-diabetics compounds in grains by germination. Crit Rev. Food Sci. Nutr..

[29] Chávez García S.N., Rodríguez-Herrera R., Nery Flores S., Silva-Belmares S.Y., Esparza-González S.C., Ascacio-Valdés J.A., Flores-Gallegos A.C. (2023). Sprouts as probiotic carriers: A new trend to improve consumer nutrition. Food Chem..

[30] Kabeer S., Govindarajan N., Radhakrishnan P., Essa M.M., Qoronfleh M.W. (2023). Effect of drying technique on physiochemical and nutritional properties of *Eleusine coracana* (finger millet) porridge powder. J. Food Sci. Technol..

[31] Bhatta S., Stevanovic Janezic T., Ratti C. (2020). Freeze-Drying of Plant-Based Foods. Foods.

[32] Privatti R.T., Capellini M.C., Thomazini M., Favaro-Trindade C.S., Rodrigues C.E.C. (2022). Profile and content of isoflavones on flaked and extruded soybeans and okara submitted to different drying methods. Food Chem..

[33] Aborus N.E., Šaponjac V.T., Čanadanović-Brunet J., Ćetković G., Hidalgo A., Vulić J., Šeregelj V. (2018). Sprouted and Freeze-Dried Wheat and Oat Seeds—Phytochemical Profile and in Vitro Biological Activities. Chem. Biodivers..

[34] Shen Y., Tang X., Li Y. (2021). Drying methods affect physicochemical and functional properties of quinoa protein isolate. Food Chem..

[35] Han C., Zhen S., Zhu G., Bian Y., Yan Y. (2017). Comparative metabolome analysis of wheat embryo and endosperm reveals the dynamic changes of metabolites during seed germination. Plant Physiol. Bioch..

[36] Zhang L., Liu X., Xu L., Xie M., Yu M. (2024). Non-Targeted Metabolomics Analysis of γ–Aminobutyric Acid Enrichment in Germinated Maize Induced by Pulsed Light. Foods.

[37] Kim H., Kim O.W., Ahn J.H., Kim B.M., Oh J., Kim H.J. (2020). Metabolomic analysis of germinated brown rice at different germination stages. Foods.

[38] Park B.I., Kim J., Lee K., Lim T., Hwang K.T. (2019). Flavonoids in common and tartary buckwheat hull extracts and antioxidant activity of the extracts against lipids in mayonnaise. J. Food. Sci. Technol..

[39] Chen Y., Zhang R., Song Y., He J., Sun J., Bai J., An Z., Dong L., Zhan Q., Abliz Z. (2009). RRLC-MS/MS-based metabonomics combined with in-depth analysis of metabolic correlation network: Finding potential biomarkers for breast cancer. Analyst.

[40] Li T., Yang Y., Wang X., Dai W., Zhang L., Piao C. (2021). Flavonoids derived from buckwheat hull can break advanced glycation end-products and improve diabetic nephropathy. Food Funct..

[41] Singh B., Singh J.P., Kaur A., Singh N. (2017). Phenolic composition and antioxidant potential of grain legume seeds: A review. Food. Res. Int..

[42] Karmann J., Müller B., Hammes U.Z. (2018). The long and winding road: Transport pathways for amino acids in Arabidopsis seeds. Plant Reprod..

[43] Kawakami K., Yamada K., Yamada T., Nabika T., Nomura M. (2018). Antihypertensive Effect of γ-Aminobutyric Acid-Enriched Brown Rice on Spontaneously Hypertensive Rats. J. Nutr. Sci. Vitaminol..

[44] Cheng Z., Qiao D., Zhao S., Zhang B., Lin Q., Xie F. (2022). Whole grain rice: Updated understanding of starch digestibility and the regulation of glucose and lipid metabolism. Compr. Rev. Food Sci. Food Saf..

[45] Borgonovi S.M., Chiarello E., Pasini F., Picone G., Marzocchi S., Capozzi F., Bordoni A., Barbiroli A., Marti A., Iametti S. (2023). Effect of sprouting on biomolecular and antioxidant features of common buckwheat (*Fagopyrum esculentum*). Foods.

[46] Ma Z., Marsolais F., Bernards M.A., Sumarah M.W., Bykova N.V., Igamberdiev A.U. (2016). Glyoxylate cycle and metabolism of organic acids in the scutellum of barley seeds during germination. Plant Sci..

[47] Niro S., D’Agostino A., Fratianni A., Cinquanta L., Panfili G. (2019). Gluten-free alternative grains: Nutritional evaluation and bioactive compounds. Foods.

[48] Tan J., McKenzie C., Potamitis M., Thorburn A.N., Mackay C.R., Macia L. (2014). The role of short-chain fatty acids in health and disease. Adv. Immunol..

[49] Wang H., Fu Y., Zhao Q., Hou D., Yang X., Bai S., Diao X., Xue Y., Shen Q. (2022). Effect of different processing methods on the millet polyphenols and their anti-diabetic potential. Front. Nutr..

[50] Hou S., Zhang Y., Zhao B., Man X., Ma G., Men Y., Du W., Yang Y., Li H., Han Y. (2022). Heterologous expression of SiFBP, a folate-binding protein from foxtail millet, confers increased folate content and altered amino acid profiles with nutritional potential to *Arabidopsis*. J. Agric. Food Chem..

[51] Shan S., Niu J., Yin R., Shi J., Zhang L., Wu C., Li H., Li Z. (2022). Peroxidase from foxtail millet bran exerts anti-colorectal cancer activity via targeting cell-surface GRP78 to inactivate STAT3 pathway. Acta Pharm. Sin. B.

[52] Weidmann A.E. (2012). Dihydroquercetin: More than just an impurity?. Eur. J. Pharmacol..

[53] Li X., Gao J., Song J., Guo K., Hou S., Wang X., He Q., Zhang Y., Zhang Y., Yang Y. (2022). Multi-omics analyses of 398 foxtail millet accessions reveal genomic regions associated with domestication, metabolite traits, and anti-inflammatory effects. Mol. Plant..

[54] Cai J., Wen H., Zhou H., Zhang D., Lan D., Liu S., Li C., Dai X., Song T., Wang X. (2023). Naringenin: A flavanone with anti-inflammatory and anti-infective properties. Biomed. Pharmacother..

[55] Sharma S., Sharma N., Handa S., Pathania S. (2017). Evaluation of health potential of nutritionally enriched Kodo millet (*Eleusine coracana*) grown in Himachal Pradesh, India. Food Chem..

[56] Javelle F., Lampit A., Bloch W., Häussermann P., Johnson S.L., Zimmer P. (2020). Effects of 5-hydroxytryptophan on distinct types of depression: A systematic review and meta-analysis. Nutr. Rev..

