# Supplementary material for: The Effect of Different Processing Methods on Metabolite Profiles by Comparative Metabolomics in Kernels and Sprouted Seeds of Foxtail Millet
Source: Foods. 2025 May 27;14(11):1900. doi: 10.3390/foods14111900 (PMC12154249; doi:10.3390/foods14111900)
Supplement: Supplementary file 1 [file foods-14-01900-s001.zip › Figures S1-S6.pdf]

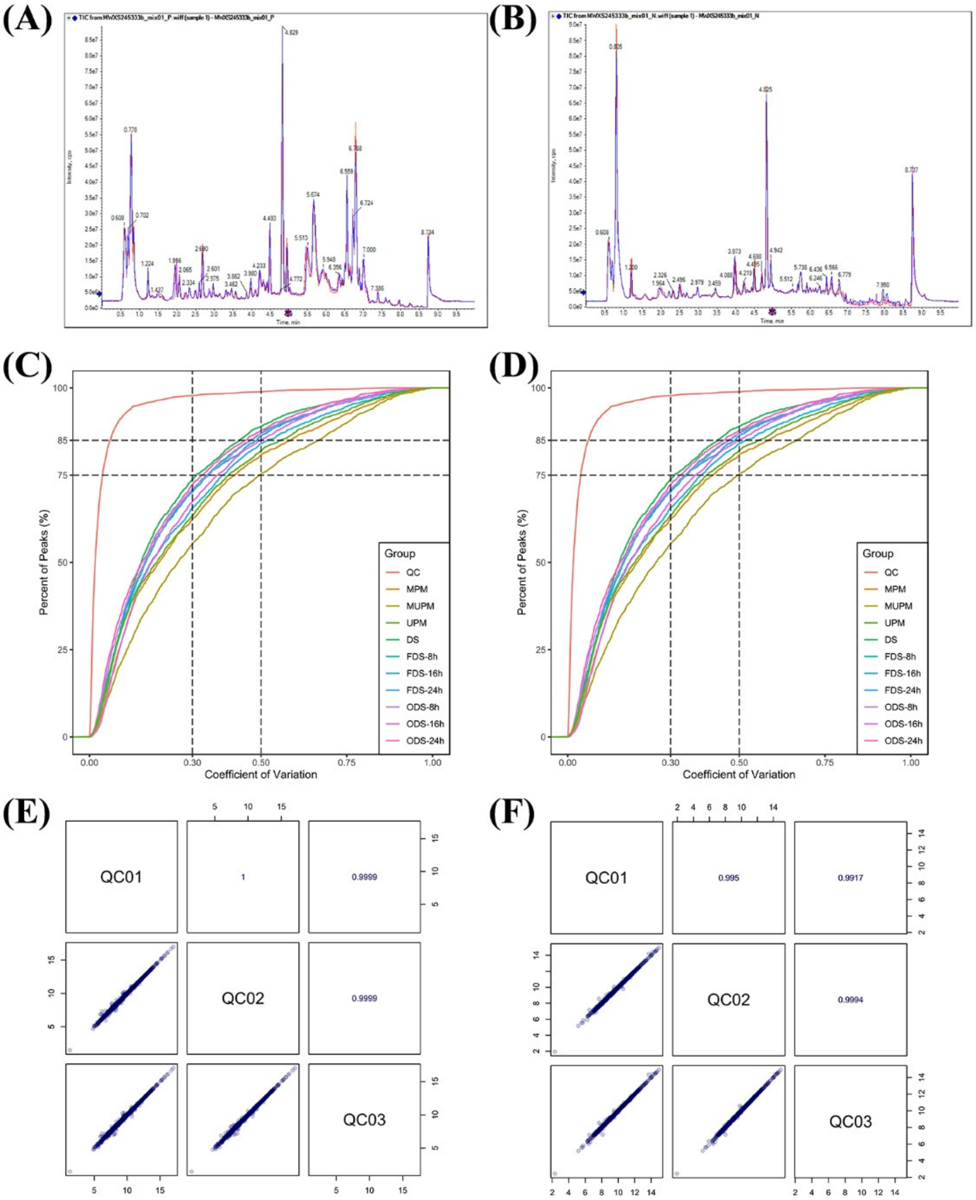

Figure S1. Quality assessment of metabolome assays. (A) the total ion current (TIC) chromatograms of the QC samples under Pos scanning mode. (B) the total ion current (TIC) chromatograms of the QC samples under Neg scanning mode. (C) Distribution map of CV value of all samples under Pos scanning mode. (D) Distribution map of CV value of all samples under Neg scanning mode. (E) Correlation analysis of QC samples under Pos scanning mode. (F) Correlation analysis of QC samples under Neg scanning mode.

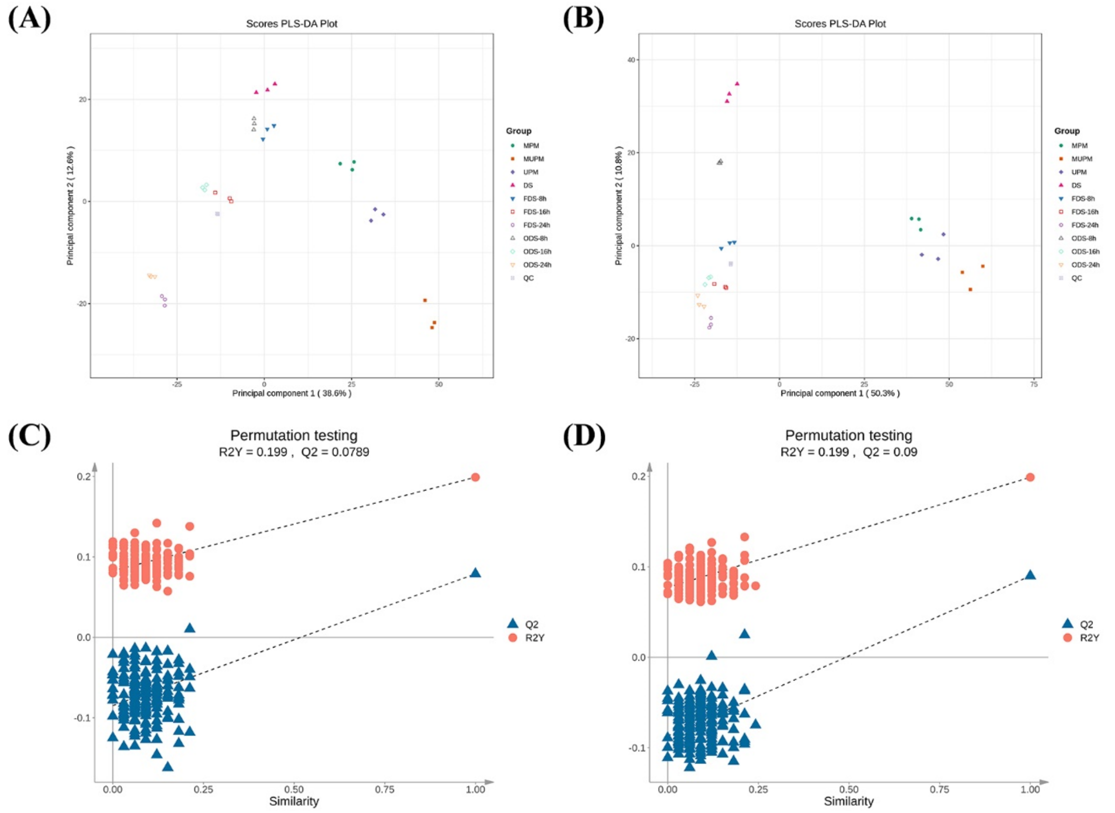

Figure S2. Partial least squares discriminant analysis under Pos and Neg modes. (A) Partial least squares discriminant analysis under Pos mode. (B) Partial least squares discriminant analysis under Neg mode. (C) Permutation testing of the partial least squares discriminant analysis model in Pos mode. (D) Permutation testing of the partial least squares discriminant analysis model in Neg mode.

**Class**	**ODS-8 h vs DS (Up | Down)**	**ODS-16 h vs DS (Up | Down)**	**ODS-24 h vs DS (Up | Down)**
Amino acids and derivatives	**7.09% (37/522)** | 6.90% (36/522)	**10.92% (57/522)** | 9.39% (49/522)	**17.05% (89/522)** | 9.39% (49/522)
Organic acids	**9.43% (23/244)** | 5.33% (13/244)	**14.34% (35/244)** | 10.25% (25/244)	**17.62% (43/244)** | 10.25% (25/244)
Benzene and substituted derivatives	5.70% (11/193) | 7.25% (14/193)	**10.36% (20/193)** | 6.74% (13/193)	**12.44% (24/193)** | 9.33% (18/193)
Alkaloids	**7.56% (9/119)** | 2.52% (3/119)	**10.08% (12/119)** | 2.52% (3/119)	**22.69% (27/119)** | 6.72% (8/119)
Lipids	0.85% (1/118) | 10.17% (12/118)	4.24% (5/118) | 9.32% (11/118)	**10.17% (12/118)** | 7.63% (9/118)
GP (Glycerophospholipid)	5.43% (5/92) | 9.78% (9/92)	9.78% (9/92) | 9.78% (9/92)	**17.39% (16/92)** | 11.96% (11/92)
Alcohol and amines	**8.24% (7/85)** | 5.88% (5/85)	**14.12% (12/85)** | 11.76% (10/85)	**10.59% (9/85)** | 8.24% (7/85)
Nucleotides and derivatives	**18.75% (15/80)** | 5.00% (4/80)	**26.25% (21/80)** | 8.75% (7/80)	**31.25% (25/80)** | 6.25% (5/80)
Phenolic acids	**6.67% (5/75)** | 5.33% (4/75)	4.00% (3/75) | 17.33% (13/75)	9.33% (7/75) | 20.00% (15/75)
Flavonoids	2.99% (2/67) | 5.97% (4/67)	8.96% (6/67) | 17.91% (12/67)	5.97% (4/67) | 25.37% (17/67)
Heterocyclic compounds	4.92% (3/61) | 4.92% (3/61)	**8.20% (5/61)** | 4.92% (3/61)	**9.84% (6/61)** | 6.56% (4/61)
Terpenoids	6.38% (3/47) | 14.89% (7/47)	10.64% (5/47) | 21.28% (10/47)	6.38% (3/47) | 17.02% (8/47)
GL (Glycerolipids)	**13.04% (6/46)** | 6.52% (3/46)	6.52% (3/46) | 13.04% (6/46)	2.17% (1/46) | 13.04% (6/46)
Lignans and Coumarins	0.00% (0/27) | 14.81% (4/27)	3.70% (1/27) | 22.22% (6/27)	11.11% (3/27) | 22.22% (6/27)
FA (Fatty Acyls)	0.00% (0/20) | 0.00% (0/20)	0.00% (0/20) | 0.00% (0/20)	0.00% (0/20) | 5.00% (1/20)
Steroids	0.00% (0/13) | 15.38% (2/13)	0.00% (0/13) | 23.08% (3/13)	0.00% (0/13) | 0.00% (0/13)
SL (Sphingolipid)	**14.29% (1/7)** | 0.00% (0/7)	0.00% (0/7) | 0.00% (0/7)	0.00% (0/7) | 0.00% (0/7)
Tannins	0.00% (0/5) | 20.00% (1/5)	40.00% (2/5) | 60.00% (3/5)	20.00% (1/5) | 40.00% (2/5)
Quinones	25.00% (1/4) | 25.00% (1/4)	0.00% (0/4) | 25.00% (1/4)	25.00% (1/4) | 50.00% (2/4)
Others	**8.85% (20/226)** | 5.75% (13/226)	**13.27% (30/226)** | 8.41% (19/226)	**16.37% (37/226)** | 10.18% (23/226)
Total	**7.26% (149/2051)** | 6.73% (138/2051)	**11.02% (226/2051)** | 9.90% (203/2051)	**15.02% (308/2051)** | 10.53% (216/2051)

Figure S3. The upregulation and downregulation ratios and quantities of different types of metabolites in ODSs. Metabolite categories with a greater number of upregulated metabolites than downregulated ones are shown in bold.

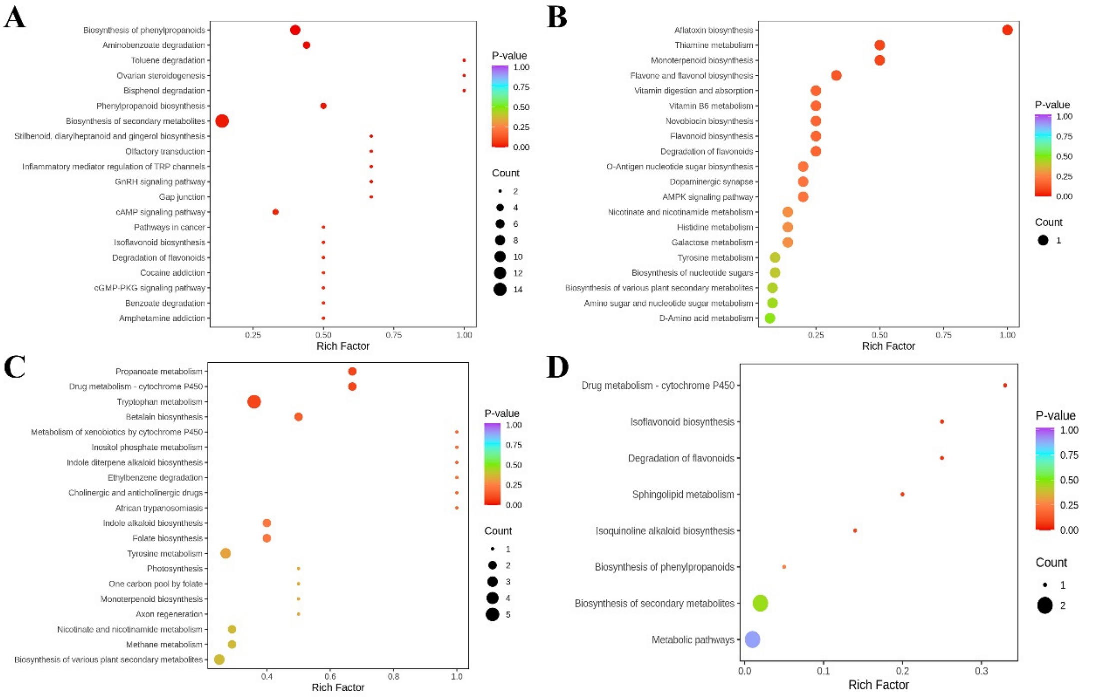

Figure S4. Original KEGG enrichment analysis results of differential metabolites in millet under different polishing methods. (A) The KEGG enrichment analysis dot plot of 210 common differential metabolites in UPM (Ultra-Polished Millet), MUPM (Manually Ultra-Polished Millet) and MPM (Manually Polished Millet) upon comparison with DS (Dry Seed). (B) The KEGG enrichment analysis dot plot of 57 metabolites with specific differential accumulation in UPM. (C) The KEGG enrichment analysis dot plot of 342 metabolites with specific differential accumulation in MUPM. (D) The KEGG enrichment analysis dot plot of 40 metabolites with specific differential accumulation in MPM.

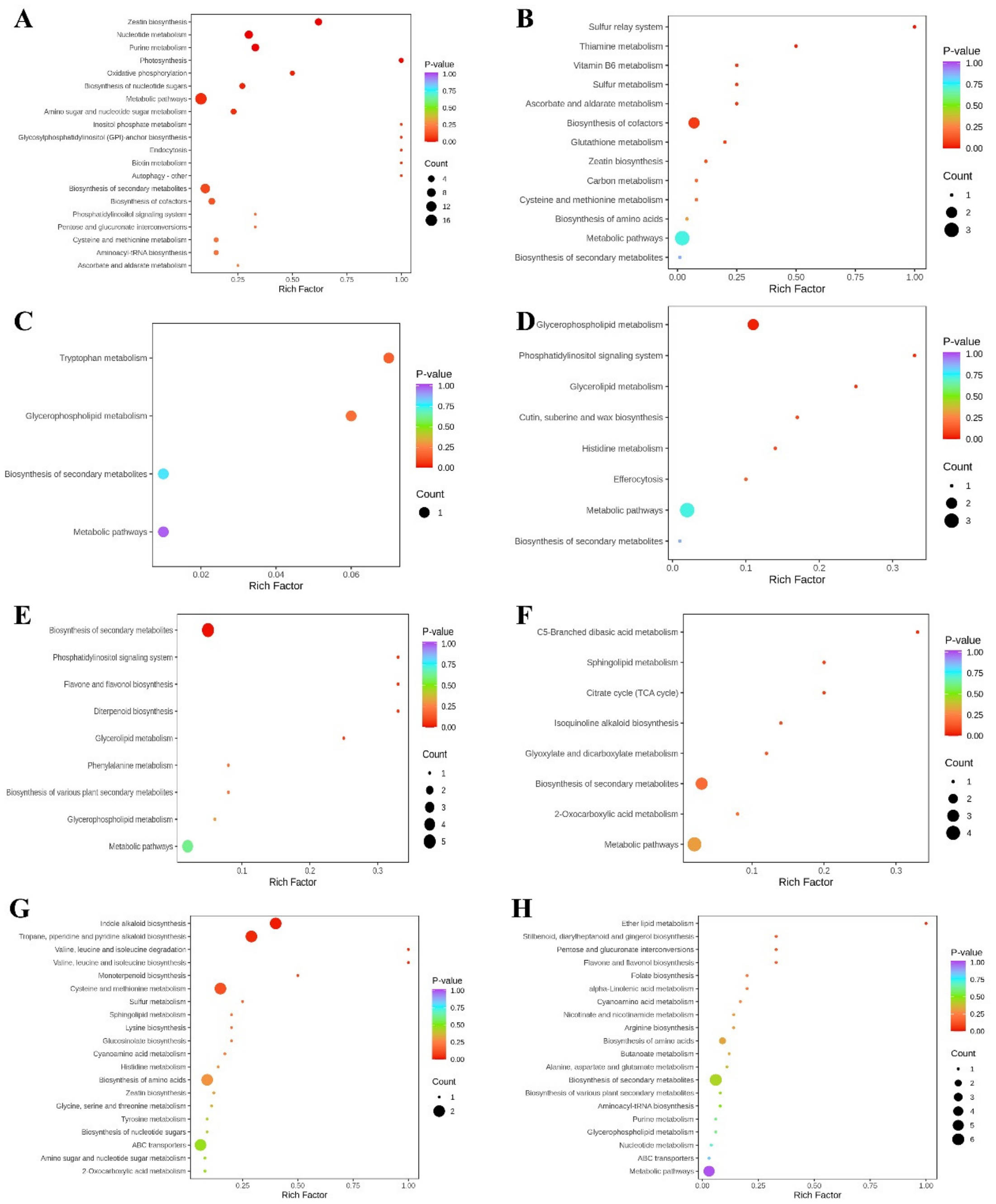

Figure S5. Original KEGG enrichment analysis results of differential metabolites in sprouted seeds at different germination stages. (A) and (B) represent dot plots of KEGG enrichment analysis for 104 upregulated and 87 downregulated metabolites in sprouted seeds at 8 h, 16 h, and 24 h compared to dry seeds, respectively. (C) and (D) represent dot plots of KEGG enrichment analysis for specifically 32 upregulated and 34 downregulated metabolites in germinating seeds at 8 h, respectively. (E) and (F) represent dot plots of KEGG enrichment analysis for specifically 28 upregulated and 23 downregulated metabolites in germinating seeds at 16 h, respectively. (G) and (H) represent dot plots of KEGG enrichment analysis for specifically 95 upregulated and 98 downregulated metabolites in germinating seeds at 24 h, respectively.

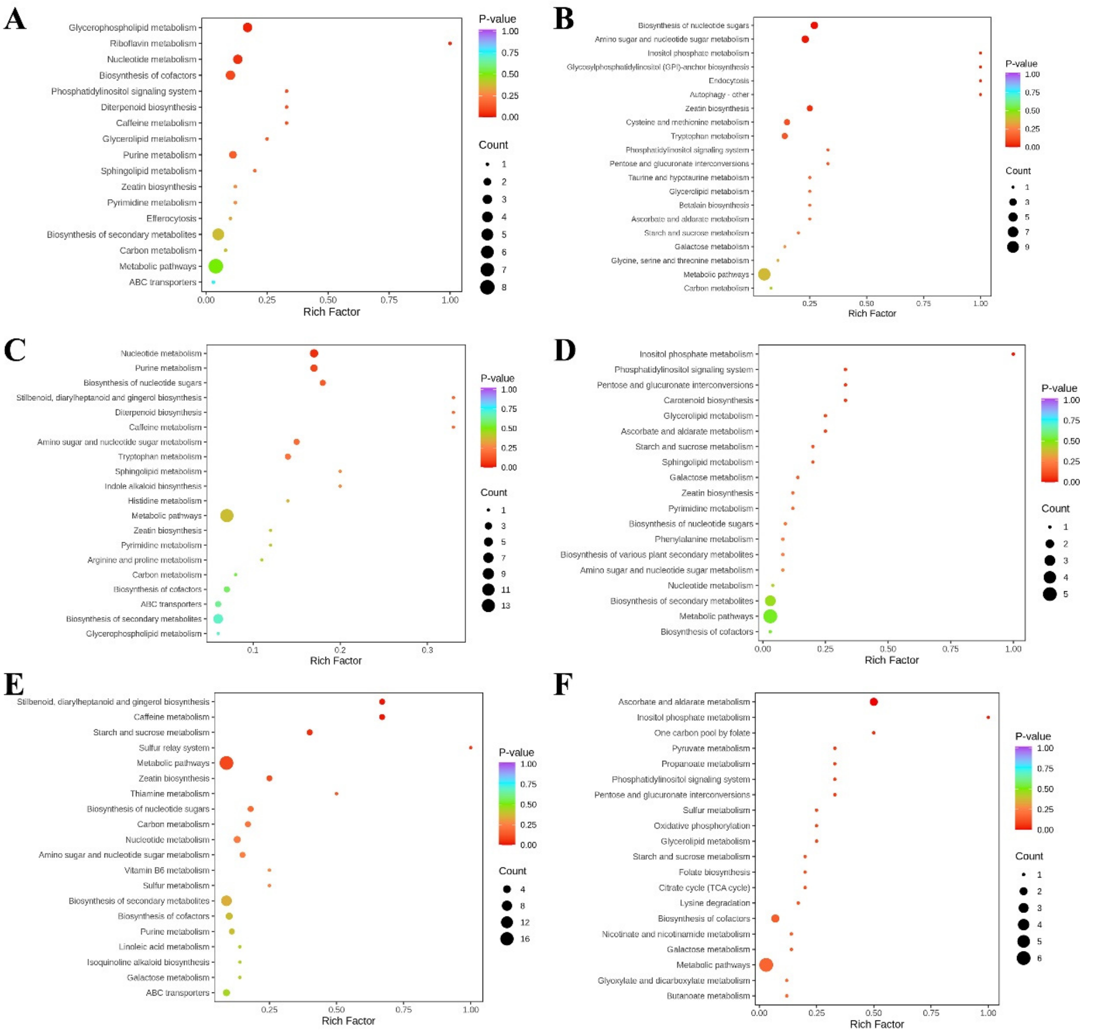

Figure S6. Original KEGG enrichment analysis results of differential metabolites in sprouted seeds with different processing methods. (A) and (B) represent KEGG enrichment dot plots of 82 upregulated and 136 downregulated metabolites, respectively, in 8-hour germinated seeds under freeze-drying and oven-drying treatments. (C) and (D) represent KEGG enrichment dot plots of 117 upregulated and 97 downregulated metabolites, respectively, in 16-hour germinated seeds under freeze-drying and oven-drying treatments. (E) and (F) represent KEGG enrichment dot plots of 134 upregulated and 69 downregulated metabolites, respectively, in 24-hour germinated seeds under freeze-drying and oven-drying treatments.
